# Supplementary material for: Weight loss practices and eating behaviours among female physique athletes: Acquiring the optimal body composition for competition
Source: PLoS One. 2022 Jan 14;17(1):e0262514. doi: 10.1371/journal.pone.0262514 (PMC8759685; doi:10.1371/journal.pone.0262514)
Supplement: Attachment — Submitted filename: Response to Reviewers.docx [file pone.0262514.s002.docx]

PONE-D-21-15998R1

Weight loss practices and eating behaviours among female physique athletes: Acquiring the optimal body composition for competition

PLOS ONE

Dear Dr. Alwan,

Thank you for submitting your manuscript to PLOS ONE. After careful consideration, we feel that it has merit but does not fully meet PLOS ONE’s publication criteria as it currently stands. Therefore, we invite you to submit a revised version of the manuscript that addresses the points raised during the review process.

Specifically, please address the minor revisions as described by reviewer #1. 

Please submit your revised manuscript by November 29, 2021. If you will need more time than this to complete your revisions, please reply to this message or contact the journal office at [plosone@plos.org](mailto:plosone@plos.org). When you're ready to submit your revision, log on to https://hes32-ctp.trendmicro.com:443/wis/clicktime/v1/query?url=https%3a%2f%2fwww.editorialmanager.com%2fpone%2f&umid=82c72bd9-6c69-4de7-8d15-5e237000c8ac&auth=e7d0e2218e6161c78f47b51039a961804123edc5-872d211937586a6a77dd5462d7a2d68c2b3cb895 and select the 'Submissions Needing Revision' folder to locate your manuscript file.

Please include the following items when submitting your revised manuscript:

- A rebuttal letter that responds to each point raised by the academic editor and reviewer(s). You should upload this letter as a separate file labeled 'Response to Reviewers'.
- A marked-up copy of your manuscript that highlights changes made to the original version. You should upload this as a separate file labeled 'Revised Manuscript with Track Changes'.
- An unmarked version of your revised paper without tracked changes. You should upload this as a separate file labeled 'Manuscript'.

If you would like to make changes to your financial disclosure, please include your updated statement in your cover letter. Guidelines for resubmitting your figure files are available below the reviewer comments at the end of this letter.

If applicable, we recommend that you deposit your laboratory protocols in protocols.io to enhance the reproducibility of your results. Protocols.io assigns your protocol its own identifier (DOI) so that it can be cited independently in the future. For instructions see: [https://journals.plos.org/plosone/s/submission-guidelines#loc-laboratory-protocols](https://hes32-ctp.trendmicro.com:443/wis/clicktime/v1/query?url=https%3a%2f%2fjournals.plos.org%2fplosone%2fs%2fsubmission%2dguidelines%23loc%2dlaboratory%2dprotocols&umid=82c72bd9-6c69-4de7-8d15-5e237000c8ac&auth=e7d0e2218e6161c78f47b51039a961804123edc5-d2b3fc86b95ec3f1b67918b17fc07c0dabab27ea). Additionally, PLOS ONE offers an option for publishing peer-reviewed Lab Protocol articles, which describe protocols hosted on protocols.io. Read more information on sharing protocols at [https://plos.org/protocols?utm_medium=editorial-email&utm_source=authorletters&utm_campaign=protocols](https://hes32-ctp.trendmicro.com:443/wis/clicktime/v1/query?url=https%3a%2f%2fplos.org%2fprotocols%3futm%5fmedium%3deditorial%2demail%26utm%5fsource%3dauthorletters%26utm%5fcampaign%3dprotocols&umid=82c72bd9-6c69-4de7-8d15-5e237000c8ac&auth=e7d0e2218e6161c78f47b51039a961804123edc5-596d8f072bec150cc2dc140ad71d0c5242429762).

We look forward to receiving your revised manuscript.

Kind regards,

Cherilyn N. McLester, PhD
Academic Editor
PLOS ONE

Journal Requirements:

Please review your reference list to ensure that it is complete and correct. If you have cited papers that have been retracted, please include the rationale for doing so in the manuscript text, or remove these references and replace them with relevant current references. Any changes to the reference list should be mentioned in the rebuttal letter that accompanies your revised manuscript. If you need to cite a retracted article, indicate the article’s retracted status in the References list and also include a citation and full reference for the retraction notice.

The reference list has been updated and checked thoroughly to meet the journal requirements. Changes have been highlighted in red in the ‘Revised manuscript with tracked changes’

Additional Editor Comments (if provided).’

[Note: HTML markup is below. Please do not edit.]

Reviewers' comments:

Reviewer's Responses to Questions

**Comments to the Author**

1. If the authors have adequately addressed your comments raised in a previous round of review and you feel that this manuscript is now acceptable for publication, you may indicate that here to bypass the “Comments to the Author” section, enter your conflict of interest statement in the “Confidential to Editor” section, and submit your "Accept" recommendation.

Reviewer #1: (No Response)

Reviewer #2: All comments have been addressed

2. Is the manuscript technically sound, and do the data support the conclusions?

The manuscript must describe a technically sound piece of scientific research with data that supports the conclusions. Experiments must have been conducted rigorously, with appropriate controls, replication, and sample sizes. The conclusions must be drawn appropriately based on the data presented.

Reviewer #1: Yes

Reviewer #2: Yes

3. Has the statistical analysis been performed appropriately and rigorously?

Reviewer #1: Yes

Reviewer #2: Yes

4. Have the authors made all data underlying the findings in their manuscript fully available?

The [PLOS Data policy](https://hes32-ctp.trendmicro.com:443/wis/clicktime/v1/query?url=http%3a%2f%2fwww.plosone.org%2fstatic%2fpolicies.action%23sharing&umid=82c72bd9-6c69-4de7-8d15-5e237000c8ac&auth=e7d0e2218e6161c78f47b51039a961804123edc5-a37b1a350ace227282564ac0b4332120802a63cb) requires authors to make all data underlying the findings described in their manuscript fully available without restriction, with rare exception (please refer to the Data Availability Statement in the manuscript PDF file). The data should be provided as part of the manuscript or its supporting information, or deposited to a public repository. For example, in addition to summary statistics, the data points behind means, medians and variance measures should be available. If there are restrictions on publicly sharing data—e.g. participant privacy or use of data from a third party—those must be specified.

Reviewer #1: No

Reviewer #2: Yes

5. Is the manuscript presented in an intelligible fashion and written in standard English?

PLOS ONE does not copyedit accepted manuscripts, so the language in submitted articles must be clear, correct, and unambiguous. Any typographical or grammatical errors should be corrected at revision, so please note any specific errors here.

Reviewer #1: Yes

Reviewer #2: Yes

6. Review Comments to the Author

Please use the space provided to explain your answers to the questions above. You may also include additional comments for the author, including concerns about dual publication, research ethics, or publication ethics. (Please upload your review as an attachment if it exceeds 20,000 characters)

Reviewer #1: Dear authors
Thank you for considering the recommendations I made in the previous review. I find the manuscript improved, and have only minor remarks:

We thank this reviewer for their extremely thorough review and are grateful for the comments that have improved this manuscript. We are pleased to hear the reviewer feels that the manuscript has improved, and we have now attempted to address further comments in hopes the reviewer now considers the revised manuscript worthy of publication.

Does «chronic gradual dieting» mean that they were always on a kcal-reduced diet, or that they gradually decreased kcal-intake during the contest dieting period? If it means the latter, I suggest to omit the word “chronic”.

We appreciate the reviewer’s line of thinking here. It is indeed the latter. We decided to omit “chronic” from weight loss practices throughout the manuscript.

Line 149-150: I suggest you also add information on the total score range for the questionnaire.

We thank the reviewer for this suggestion. Unfortunately, we do not have this information as it is not provided in the original paper by Artioli et al., (2010).

RWLS is based on scores from weight and diet history and the weight loss behaviours questions. As an example, in the weight and diet history questions, athletes will receive 1 point per kg of weight they lose, while on the weight behaviours questions, answers will be scored between 0-4. As such, it is very difficult to provide a precise range. However, we have reported: “The RWLQ scoring system indicates the higher the score obtained, the more aggressive the weight loss behaviours” on line 149-150.

Table 2: please add similar explanation on what numbers represent, as done in table 1 (and I suggest you add this to the header/the text in the title of the table): “Values are presented as mean ± SD and include the range in brackets”.

We would like to thank the reviewer for bringing this to our attention. This has now been corrected. “Values are presented as mean ± SD and include the range in brackets” has been added in the title of the table 2 on line 221. We have also replicated this explanation in table title of table 1 on line 196-197 for consistency.

Line 283: Laxative use is mentioned as a frequent pathogenic weight control method, however the relevant question in the EAT-26 questionnaire asks about laxatives, diet pills and diuretics in the same question. As such it would be more precise to mention all these methods in the relevant section of the text and figure 3, including in the discussion. Laxative use is previously found to be less frequently used (previous mentioned references), however diet pills and diuretics are more frequently used (the latter also being anecdotally/taken from personal insight to the sport). This is underscored by the finding you specifically mention in the discussion line 350-352.

Thank you for noting our error in reporting. This has now been corrected in the results, discussion, figure 3 title, and the y-axis on the figure 3.

For example, this has been corrected on line 282-283: “For the combined group, 42.4% used two out of three PWCM (laxatives, diet pills and diuretics, and binge eating), while 13.3% used all methods to manage their weight. Laxatives, diet pills and diuretics….. “

Paragraph initiated from line 374: here shortly addressing whether the EAT-26 is valid to capture DE-risk in physique athletes is recommended (I notice this has been addressed in the “limitation” of the paper, however I find It reasonable to shortly address here too, to quickly draw attention to such a dilemma).

We thank the reviewer for this comment and agree with this point. We have included: “Although, EAT-26 questionnaire has been validated in other athletic groups, further work to validate it in this specific population is necessary” on line 389-391 to acknowledge this issue.

We have now also included this additional detail in the methods section of the study design on line 120 where we directly talk about using the EAT-26 questionnaire.

Paragraph from line 386: First, the explanation for discrepancies presented in line 389 needs clarification (I personally did not automatically understand what the authors meant).

Second; I find the most obvious reason for the discrepancies between the studies that there are different sexes in the two studies (Pickard studied male bodybuilders, you study female physique athletes). Taking the items in the EAT-26 into consideration, it is not very typical for a male body builder to respond positive to the questions in the relevant questionnaire (they are not preoccupied of being thinner, or afraid of eating/afraid of sugar or food. Not even on when being on a diet). I understand that you find it interesting to compare to a study which have used the same questionnaire, however, I find it difficult or at least less relevant, as there are obvious reasons to why there are discrepancies in your finding (type of sport i.e. bodybuilding versus fitness sports, sexes included, a questionnaire that not very well captures the issue at quest within these type of sports).

We thank the reviewer for raising this point. We agree that a better study could be used to make comparisons to here. We have now removed these sentences from the manuscript and have included a recent study on line 376 (Money-Taylor et al., 2021) which recruited female physique athletes and used a similar screening tool to our study.

Thank you.

Reviewer #2: Thank you to the authors for addressing all of my previous comments. Well done on the preparation of this manuscript.

We thank this reviewer for their thorough and constructive review and are grateful for the comments that have improved this manuscript.

7. PLOS authors have the option to publish the peer review history of their article ([what does this mean?](https://hes32-ctp.trendmicro.com:443/wis/clicktime/v1/query?url=https%3a%2f%2fjournals.plos.org%2fplosone%2fs%2feditorial%2dand%2dpeer%2dreview%2dprocess%23loc%2dpeer%2dreview%2dhistory&umid=82c72bd9-6c69-4de7-8d15-5e237000c8ac&auth=e7d0e2218e6161c78f47b51039a961804123edc5-fa84709443f20b00514d9881c75fc339f4388838)). If published, this will include your full peer review and any attached files.


If you choose “no”, your identity will remain anonymous but your review may still be made public.


**Do you want your identity to be public for this peer review?** For information about this choice, including consent withdrawal, please see our [Privacy Policy](https://hes32-ctp.trendmicro.com:443/wis/clicktime/v1/query?url=https%3a%2f%2fwww.plos.org%2fprivacy%2dpolicy&umid=82c72bd9-6c69-4de7-8d15-5e237000c8ac&auth=e7d0e2218e6161c78f47b51039a961804123edc5-3ba10b523cf53dc57a74d42394f1e351d4b94c34).

Reviewer #1: No

Reviewer #2: **Yes:** Lachlan Mitchell

[NOTE: If reviewer comments were submitted as an attachment file, they will be attached to this email and accessible via the submission site. Please log into your account, locate the manuscript record, and check for the action link "View Attachments". If this link does not appear, there are no attachment files.]

While revising your submission, please upload your figure files to the Preflight Analysis and Conversion Engine (PACE) digital diagnostic tool, <https://hes32-ctp.trendmicro.com:443/wis/clicktime/v1/query?url=https%3a%2f%2fpacev2.apexcovantage.com&umid=82c72bd9-6c69-4de7-8d15-5e237000c8ac&auth=e7d0e2218e6161c78f47b51039a961804123edc5-905603a5bceedbfc166587a2a97b83cf6cc9f144>. PACE helps ensure that figures meet PLOS requirements. To use PACE, you must first register as a user. Registration is free. Then, login and navigate to the UPLOAD tab, where you will find detailed instructions on how to use the tool. If you encounter any issues or have any questions when using PACE, please email PLOS at [figures@plos.org](mailto:figures@plos.org). Please note that Supporting Information files do not need this step.

*In compliance with data protection regulations, you may request that we remove your personal registration details at any time.* [*(Remove my information/details)*](https://hes32-ctp.trendmicro.com:443/wis/clicktime/v1/query?url=https%3a%2f%2fwww.editorialmanager.com%2fpone%2flogin.asp%3fa%3dr&umid=82c72bd9-6c69-4de7-8d15-5e237000c8ac&auth=e7d0e2218e6161c78f47b51039a961804123edc5-8b25cce28c31ffb54382b6f68604dfc79ab385cc)*. Please contact the publication office if you have any questions.*
